# Lack of relationship between 25-hydoxyvitamin D concentration and a titer of antibodies to hepatitis B surface antigen in children under 12 years of age

**DOI:** 10.1371/journal.pone.0277473

**Published:** 2022-11-10

**Authors:** Nel Dabrowska-Leonik, Jolanta Sawicka-Powierza, Ewa Bernatowska, Malgorzata Pac, Katarzyna Bernat-Sitarz, Edyta Heropolitanska-Pliszka, Barbara Pietrucha, Beata WolskaKusnierz, Aleksandra Lewandowicz-Uszynska, Bozena Mikoluc

**Affiliations:** 1 Immunology Department, Children’s Memorial Health Institute, Warsaw, Poland; 2 Department of Family Medicine, Medical University of Bialystok, Bialystok, Poland; 3 Third Department and Clinic of Pediatrics, Immunology and Rheumatology of Developmental Age, Wroclaw Medical University, Wroclaw, Poland; 4 Department of Pediatrics, Rheumatology, Immunology and Metabolic Bone Diseases, Bialystok, Poland; Institute for clinical Virology, Giessen, UNITED STATES

## Abstract

The effect of vitamin D levels on the response to the hepatitis B vaccine in childhood and the induced levels of antibodies against the hepatitis B surface antigen (anti-HBs) is not yet well understood. The study aimed to investigate the relationship between age, serum 25-hydroxyvitamin D (25(OH)D) concentration and anti-HBs titer among children under 12 years old. Serum 25(OH)D concentration and anti-HBs titer were determined in 352 healthy Caucasian children with the average age of 4.2 (2.5; 6.3) years. All children were vaccinated with 3 doses of hepatitis B vaccine (Engerix-B, GlaxoSmithKline Pharmaceuticals Limited) in infancy according to the Centers for Disease Control and Prevention recommendations. Only 14.5% of children had an optimal concentration of 25(OH)D ≥ 30 ng/mL and 71.9% children had a seroprotective anti-HBs titer ≥ 10 mIU/mL. Significant negative correlations were found between 25(OH)D, anti-HBs titer and age (*r* = –0.420, *p* = 0.000; *r* = –0.425, *p* = 0.000, respectively), and a weak positive correlation between 25(OH)D concentration and anti-HBs titer (*r* = 0.243, *p* = 0.000). Analysis of six clusters of children demonstrated that age is the main factor affecting anti-HBs titer. One third of children under 12 years of age had nonprotective anti-HBs titer < 10 mIU/mL and around 40% had vitamin D deficiency. We conclude that vitamin D status has no impact on anti-HBs titer in children vaccinated against hepatitis B virus in infancy. Age, so time since the receipt of the last dose of hepatitis B vaccine, is the main factor influencing a decline in anti-HBs titer.

## Introduction

The implementation of universal vaccination programmes and vitamin D supplementation with the aim of preventing rickets constitutes the most important preventive health measure. The first inactivated hepatitis B (HB) vaccine was licensed for use in the United States of America in November 1981 [1] and France in 1982 [2], while a recombinant vaccine was included in universal immunisation programmes in the last decade of the 20th century, thus decreasing incidence and mortality resulting from an acute or fulminant course of HB [3]. In Poland, in 1989, mandatory vaccination against HB virus (HBV) was introduced in newborns born to HBV positive mothers. Since 1994, all newborns have been vaccinated in several regions of Poland, and since 1996, all newborns have been vaccinated as part of the Polish mandatory immunisation schedule [4].

According to the Centers for Disease Control and Prevention (CDC), a seroprotective level against HBV infection is defined as having antibodies to hepatitis B surface antigen (anti-HBs) titer ≥ 10mIU/mL when measured 1–2 months after administration of a complete immunisation course in immunocompetent children and adults [5]. Literature data indicate that in 15–50% of children who reach anti-HBs titer above 10 mIU/mL in response to primary vaccination, these levels drop below 10 mUI/mL within 5–10 years of immunisation [6]. The beneficial effect of vaccination on the epidemiology of HBV infection and immunological memory determines that resistance to the HBV lasts longer than protective antibody concentration, and therefore the administration of booster vaccination to a healthy population is not recommended [7,8]. The magnitude of vaccine response depends on genetic and external factors (e.g. preterm, term or post-term delivery, birth weight, age, immunisation schedule) [9,10], chronic diseases [11–13] and immune status [14]. The influence of diet and vitamin supplementation on antibody titer following HB vaccination is also discussed in the available literature [15].

Vitamin D is one of the factors that exerts a positive impact on vaccine response [16]. First reports on the extraskeletal effects of vitamin D and its effect on the immune system come from the 1980s [17], and the potential impact of vitamin D as an adjuvant on vaccine response is reported by a number of authors [16]. Vitamin D, through its active metabolite calcitriol-1,25-dihydroxyvitamin D (1.25(OH)2D), impacts the immune system. Antigen-presenting cells express vitamin D receptor as well as 1α-hydroxylase, an enzyme converting inactive 25-hydroxyvitamin D (25(OH)D) into active 1,25(OH)2D, causing locally high levels of calcitriol independently from the kidney enzyme [16,18]. Laboratory studies have confirmed the effect of vitamin D on innate and acquired immunity including a shift in T-cell response towards T-helper 2 [16].

Studies on the effect of vitamin D on anti-HBs titer following vaccination have produced contradictory results, suggesting a beneficial effect or no effect. The aim of our study was to evaluate the relationship between age, 25(OH)D concentration and anti-HBs titer in healthy children under 12 years of age following the administration of three doses of recombinant HB vaccine in accordance with the CDC and Polish recommended immunisation schedule of 0, 1 and 6 months.

## Materials and methods

### Study participants

Out of 524 Caucasian children who presented at the Immunology Outpatient Clinic of the Children’s Memorial Health Institute in Warsaw with suspected immune disorders from October 2017 to April 2018, 352 healthy children (208 male and 144 female) in whom immune disorders had been excluded were enrolled in the study.

Exclusion criteria were as follows: any chronic disease and use of any medication including medication containing vitamin D except for children under 3 years of age who received vitamin D at doses recommended for their age (400–600 IU/day) [19], and anti-HBs titer above 1000 mIU/mL.

Inclusion criteria for the cross-sectional study were as follows: full term delivery, good health, negative markers of inflammation (C-reactive protein; leucocytosis; erythrocyte sedimentation rate), absence of chronic diseases, not taking any medication including medication containing vitamin D except for children under 3 years of age. All children were vaccinated with three 10 μg doses of a recombinant hepatitis B vaccine (Engerix-B, GlaxoSmithKline Pharmaceuticals Limited) intramuscularly in accordance with the CDC recommended immunisation schedule at 0, 1 and 6 months.

Parents or legal guardians of all study participants were required to sign written informed consent forms which explained the aims, design and expected outcomes of the study. The study was performed in accordance with the Declaration of Helsinki on Biomedical Research Involving Human Subjects. The study was approved by the Bioethics Committee at the Children’s Memorial Health Institute (No. 20/KBE/2017).

### Measurements

Fasting blood was collected from all study participants during months with low sunlight exposure to prevent 25(OH)D concentration from being affected by solar radiation.

Total serum concentration of 25(OH)D [(25(OH)D2 and 25(OH)D3)] was determined using an automatic method based on chemiluminescent immunoassay LIAISON^®^ 25 OH Vitamin D Total (DiaSorin, Saluggia, Italy) controlled and certified by the Vitamin D Standardization and Certification Program (VDSCP) with the coefficient of variation ≤ 4.0 ng/mL. Vitamin D deficiency was confirmed at < 20 ng/mL, suboptimal concentration at 20–30 ng/mL, optimal concentration at 30–50 ng/mL and high concentration at 50–100 ng/mL [20].

Anti-HBs titer was determined by the chemiluminescent immunoassay ARCHITECT Anti-HBs (Abbott Laboratories, Sligo, Ireland) with the range of quantitation 0–1000 reported as milli-international units per milliliter (mIU/mL) according to the World Health Organization (WHO) international reference standard [21]. Three groups of subjects were identified, i.e. ‘negative’, with low anti-HBs titer < 10 mIU/mL, ‘low-positive’ with high anti-HBs titer 10–100 mIU/mL, and ‘positive’ with very high anti-HBs titer > 100 mIU/mL.

### Statistical methods

Statistical analysis was performed using the STATISTICA version 13 program. For qualitative characteristics (subgroups of gender, age, range of 25(OH)D concentration and anti-HBs titer), numbers (n) and percentages (%) were provided in the tables. Due to a very strong positive asymmetry of anti-HBs titer (values ranging from 0 to 1000 mIU/mL) as well as significant deviation from the normal distribution for age and 25(OH)D concentration, statistical description includes medians and quartiles (Q1; Q3). The Mann-Whitney *U* test was used to compare the levels of the analysed measurable features between two subgroups, including pairs of clusters. Pearson’s chi-square test was used to assess the significance of interdependence between qualitative variables. Correlations between measurable features were evaluated using Spearman’s rank correlation coefficient. The significance of the coefficient was assessed by the t-Student test.

For the assessment of the combined effect of age and 25(OH)D concentration on anti-HBs titer, six clusters, as distant from each other as possible and internally coherent, were generated by the cluster k-means method. The average of age, 25(OH)D concentration and anti-HBs titer between the clusters were compared. Differences in the levels of the analysed variables, interdependence and correlation were considered statistically significant at *p* < 0.05.

## Results

Three hundred and fifty-two children with a median (Q1; Q3) age of 4.2 (2.5; 6.3) years were enrolled into the study. The basic characteristics of study participants and comparison of the values of studied features between boys and girls are presented in Table 1. There were no significant differences in the tested variables between the sexes.

**Table 1:** Basic characteristic of study participants and comparison of tested parameters between boys and girls.

Features	All participants	Boys	Girls	*p* Values
Number, n	352	208	144	
Age, years	4.2 (2.5; 6.3)	4.3 (2.5; 6.4)	4.1 (2.5; 6.2)	0.608
**Age groups (years), n (%)**				
0.7–2	51 (14.5)	30 (14.4)	21 (14.6)	
3–5	165 (46.9)	91 (43.8)	74 (51.4)	0.301
6–11	136 (38.6)	87 (41.8))	49 (34.0)	
25(OH)D, ng/mL	22.3 (16.9; 27.0)	22.3 (16.8; 26.9)	22.2 (17.4; 27.2)	0.802
**Range of 25(OH)D concentration (ng/mL), n (%)**				
< 20	141 (40.1)	82 (39.4)	59 (41.0)	
[20–30)	160 (45.4)	95 (45.7)	65 (45.1)	0.943
≥ 30	51 (14.5)	31 (14.9)	20 (13.9)	
Anti-HBs titer, mIU/mL	30.8 (7.4; 100.5)	30.8 (7.1; 122.3)	29.9 (8.2; 84.2)	0.455
**Range of anti-HBs titer (mIU/mL), n (%)**				
< 10	99 (28.1)	58 (27.9)	41 (28.5)	
[10–100]	165 (46.9)	93 (44.7)	72 (50.0)	0.429
(100–1000]	88 (25.0)	57 (27.4)	31 (21.5)	

**Notes:** Results are presented as medians and quartiles (Q1; Q3) or numbers (n) and percentages (%). To convert values for 25-hydroxyvitamin D to nmol/L, multiply by 2.5.

**Abbreviations:** Anti-HBs, antibodies against the surface antigen of the hepatitis B virus (HBsAg); 25(OH)D, 25-hydroxyvitamin D.

In the study group, only 51 (14.5%) out of the 352 studied children had an optimal concentration of 25(OH)D ≥ 30 ng/mL. The subgroup comprised 17 (4.8%) children aged 0.7–2 years, 27 (7.7%) children aged 3–5 years and 7 (2%) children aged 6–11 years. The concentration of vitamin 25(OH)D in the range [20–30) ng/mL was found in 22 (6.3%) children aged 0.7–2 years, in 80 (22.7%) children aged 3–5 years and in 58 (16.5%) aged 6–11 years. The concentrations of vitamin 25(OH)D < 20 ng/mL were found in 12 (3.4%) children from the 0.7–2 years of age subgroup, in 58 (16.5%) children from the 3–5 years of age subgroup and in 71 (20.1%) children from the 6–11 years of age subgroup (p = 0.000).

Two hundred and fifty-three (71.9%) children had a seroprotective anti-HBs titer ≥ 10 mIU/mL. 165 (46.9%) children had a high anti-HBs titer of [10–100] mIU/mL and 88 (25%) children had a very high anti-HBs titer > 100 mIU/mL. Ninety-nine (28.1%) study participants had a low anti-HBs titer < 10 mIU/mL, including 4 children with an undetectable anti-HBs titer (2 girls and 2 boys over 3 years old). The percentages of subjects depending on age with low, high and very high levels of anti-HBs are presented in Table 2.

**Table 2:** Percentage of subjects depending on age with low, high and very high anti-HBs titer.

Age subgroups, number	Anti-HBs titer			p Values
	< 10 mIU/mL	[10–100] mIU/mL	(100–1000) mIU/mL	
0.7–2 years, n = 51	1 (2.0%)	24 (47.0%)	26 (26.0%)	
3–5 years, n = 165	44 (26.7%)	78 (47.3%)	43 (26.0%)	0.000
6–11 years, n = 136	54 (39.7%)	63 (46.3%)	19 (14.0%)	

**Abbreviations:** Anti-HBs, antibodies against the surface antigen of hepatitis B virus (HBsAg).

Statistically significant negative correlations between 25(OH)D concentration and age, and between anti-HBs titer and age were established (*r* = –0.420, *p* = 0.000; *r* = –0.425, *p* = 0.000, respectively). Additionally, a weak positive correlation between 25(OH)D concentration and anti-HBs titer (*r* = 0.243, *p* = 0.000) was found.

Comparisons of the levels of tested parameters between subgroups of children according to age subgroup (0.7–2/3–5/6–11 years) and the range of 25(OH)D concentration (<20/[20–30)/≥ 30 ng/mL) are shown in Table 3.

**Table 3:** Comparison of examined features in subgroups according to age and the range of 25(OH)D concentration.

Features	Age subgroups, years				*p* Values	
	**0.7**–**2 (I)**	**3**–**5 (II)**	**6**–**11 (III)**	**I *vs* II**	**I *vs* III**	**II *vs* III**
Number, n	51	165	136			
25(OH)D, ng/mL	26.4 (21.9; 34.0)	22.9 (18.2; 27.5)	19.0 (14.4; 23.8)	0.003	0.000	0.001
Anti-HBs titer, mIU/mL	108.4 (61.3; 354.4)	26.5 (9.1; 110.9)	14.2 (3.6; 50.6)	0.000	0.000	0.007
	**Range of 25(OH)D concentration, ng/mL**					
	**< 20 (1)**	**[20–30) (2)**	**≥ 30 (3)**	**1 *vs* 2**	**1 *vs* 3**	**2 *vs* 3**
Number, n	141	160	51			
Anti-HBs titer, mIU/mL	21.6 (5.2; 71.2)	38.0 (9.3; 127.7)	48.8 (12.4; 190.5)	0.005	0.003	0.770

**Notes:** The results are presented as medians and quartiles (Q1; Q3) or numbers (n).

**Abbreviations:** Anti-HBs, antibodies against the surface antigen of the hepatitis B virus (HBsAg); 25(OH)D, 25-hydroxyvitamin D.

The highest average 25(OH)D concentration and anti-HBs titer were found in children under 3 years of age (subgroup I) who received vitamin D supplementation. In children aged 3–5 years (subgroup II), average 25(OH)D concentration and anti-HBs titer were significantly lower compared to subgroup I, but significantly higher in comparison to children aged 6–11 years (subgroup III). This is in line with the general knowledge that anti-HBs titers decline steadily after vaccination unless a booster dose (by infection or vaccination) is present.

In children with 25(OH)D concentration < 20 ng/mL (subgroup 1), the average anti-HBs titer was significantly lower in comparison to subgroups 2 and 3, with a suboptimal and optimal concentration of 25(OH)D. The highest average anti-HBs titer was found in children with 25(OH)D concentration ≥ 30 ng/mL (subgroup 3) and it was comparable with the average anti-HBs titer in children with 25(OH)D concentration of [20–30) ng/mL (subgroup 2).

**Fig 1:**
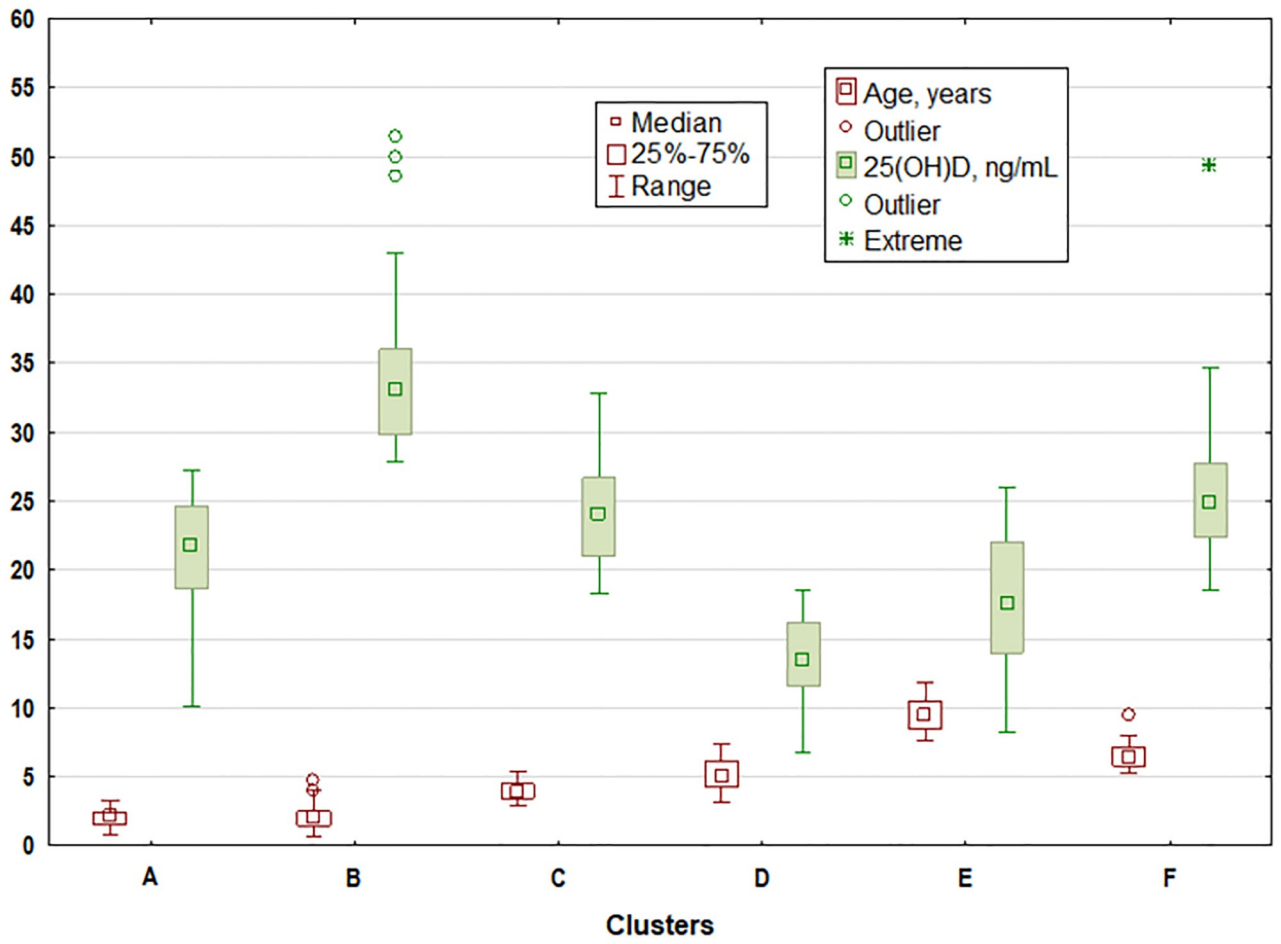
Average of age and 25(OH)D concentration (independent variables) in individual clusters of children.

**Fig 2:**
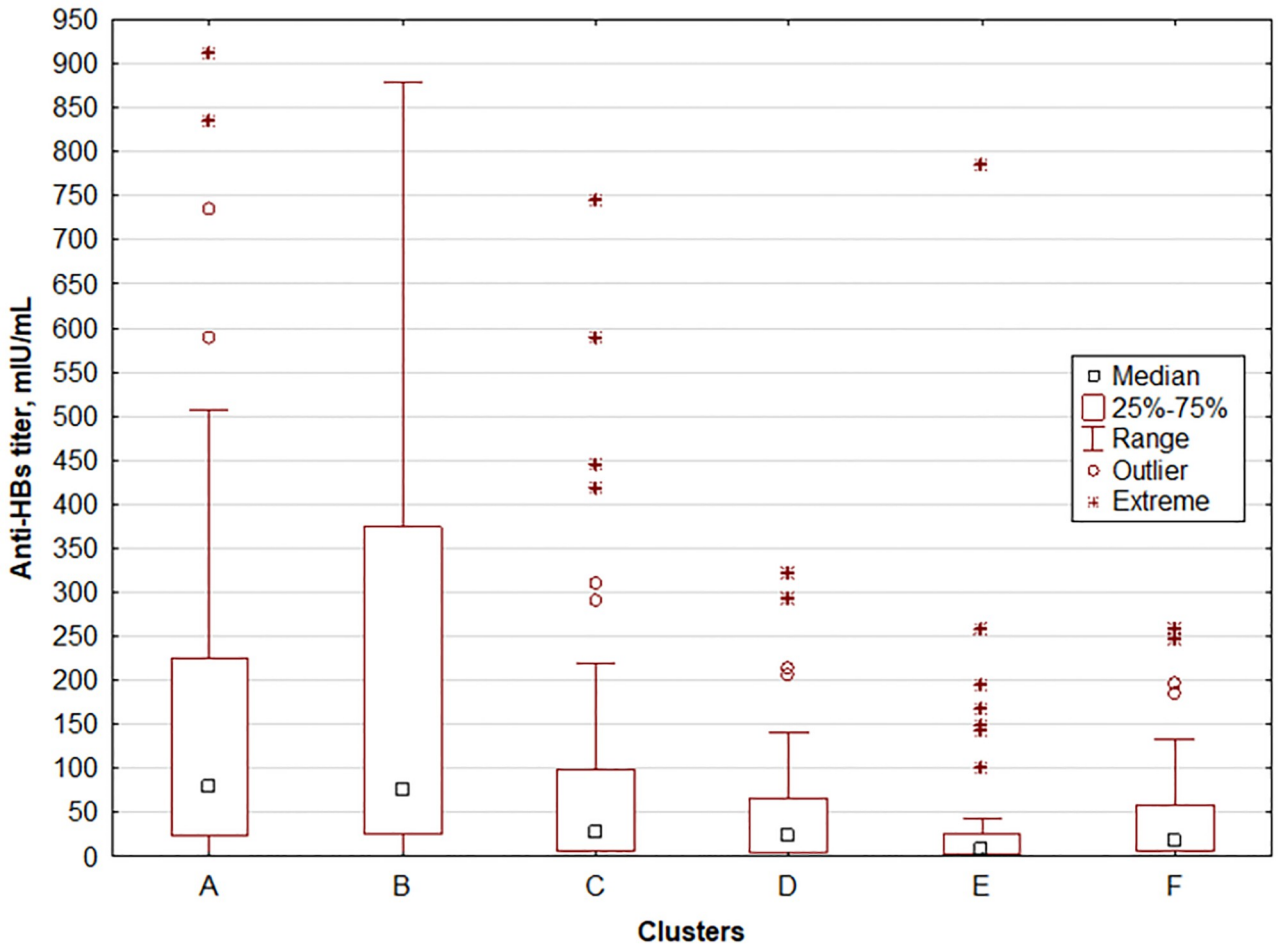
Average anti-HBs titer (dependent variable) in individual clusters of children.

To examine the combined effect of age and 25(OH)D concentration (independent variables) on anti-HBs titer (dependent variable) in children, six clusters, as distant from each other as possible and internally coherent, were generated. They are shown in Table 4 and Figs 1 and 2.

**Table 4:** Average age, 25(OH)D concentration and anti-HBs titer in six clusters of children.

Cluster	Number, n (%)	Age, years	25(OH)D, ng/mL	Anti-HBs titer, mIU/mL
A	59 (16.8)	2.2 (1.5; 2.4)	21.8 (18.7; 24.6)	79.8 (23.7; 224.7)
B	56 (15.9)	2.0 (1.5; 2.5)	33.2 (29.9; 36.0)	74.6 (25.7; 374.5)
C	70 (19.9)	3.9 (3.5; 4.5)	24.0 (21.0; 26.7)	26.8 (6.9; 98.6)
D	73 (20.7)	5.0 (4.3; 6.2)	13.5 (11.6; 16.2)	22.5 (4.1; 67.2)
E	46 (13.1)	9.4 (8.5; 10.4)	17.6 (14.0; 22.0)	8.1 (2.5; 26.7)
F	48 (13.6)	6.3 (5.8; 7.2)	24.9 (22.4; 27.7)	17.9 (6.0; 58.5)

**Notes:** The results are presented as medians and quartiles (Q1; Q3).

The significance of differences in age, 25(OH)D concentration and anti-HBs titer between pairs of clusters are presented in Table 5.

**Table 5:** *P* values for comparisons of examined variables between clusters of children.

Clusters		Variables	
	Age, years	25(OH)D, ng/mL	Anti-HBs titer, mIU/mL
		*p* Values	
Cluster A vs B	0.891	0.000	0.660
Cluster A vs C	0.000	0.000	0.000
Cluster A vs D	0.000	0.000	0.000
Cluster A vs E	0.000	0.000	0.000
Cluster A vs F	0.000	0.000	0.000
Cluster B vs C	0.000	0.000	0.002
Cluster B vs D	0.000	0.000	0.000
Cluster B vs E	0.000	0.000	0.000
Cluster B vs F	0.000	0.000	0.000
Cluster C vs D	0.000	0.000	0.266
Cluster C vs E	0.000	0.000	0.002
Cluster C vs F	0.000	0.098	0.297
Cluster D vs E	0.000	0.000	0.065
Cluster D vs F	0.000	0.000	0.927
Cluster E vs F	0.000	0.000	0.044

**Abbreviations:** Anti-HBs, antibodies against the surface antigen of the hepatitis B virus (HBsAg); 25(OH)D, 25-hydroxyvitamin D.

In cluster analysis, we considered a younger age and a higher level of vitamin D as positive factors in terms of the effect on anti-HBs titer, and an older age and a lower level of vitamin D as negative factors. The comparison of paired clusters using the Mann-Whitney *U* test showed that pair A *vs* B differed significantly only in the concentration of 25(OH)D, and pair C *vs* F differed significantly only in age. The remaining pairs differed significantly in both age and 25(OH)D concentration (p < 0.0001).

The average anti-HBs titer did not differ significantly between clusters A and B (p = 0.660), C and D (p = 0.266), C and F (p = 0.297), D and E (p = 0.065), and D and F (p = 0.927). In the remaining 10 pairs of clusters, the differences were significant.

## Discussion

In all study participants, the average vitamin D concentration was suboptimal (22.3 ng/mL), and declined with age. Only 14.5% of children reached optimal vitamin D values ≥ 30 ng/mL, while 85.5% had a deficient (40.1%) or suboptimal (45.4%) concentration. Children under 3 years old supplemented with vitamin D had a significantly higher average of vitamin D concentration in comparison to older children, who did not receive vitamin D supplementation. Among 51 children aged 0.7–2 years, only 17 (4.8%) reached optimal values of vitamin D ≥ 30 ng/mL, 12 (3.4%) had deficiency (< 20 ng/mL) and 6.3% had a suboptimal concentration.

Studies in Europe have demonstrated that 40.4% of children and adults have a serum 25(OH)D level < 50 nmol/L (20 ng/mL), and 13% have a level < 30 nmol/L (12 ng/mL). Furthermore, vitamin D deficiency has been revealed in children 4–7% aged 1–6 years old, 1–8% aged 7–14 years old, and 12–40% aged 15–18 years old [22]. Among Polish children aged 9–13 years old, 84.2% had a level of 25(OH)D < 20 ng/mL, whereas 20.2% had less than 12.5 ng/mL [23]. In a prospective study conducted among Polish infants supplemented with vitamin D, serum concentration decreased from 40.2 ± 18.8 ng/mL at 6 months to 32.0 ± 12.7 ng/mL at 12 months of life [24]. Supplementation at the recommended levels did not result in our patients under the age of 3 achieving the optimal concentration of 25(OH)D. The data presented above and our study results indicate that vitamin D supplementation is necessary in the under 12 age.

Our study demonstrated that the administration of three doses of HB vaccine in infancy evoked a seroprotective anti-HBs titer ≥ 10 mIU/mL in 71.9% of healthy children, showing no sexual predilection. The percentages of seronegative children were 2.0% aged 0.7–2 years, 26.7% aged 3–5 years and 39.7% aged 6–11 years.

Literature data indicate that the proportion of individuals with seroprotective anti-HBs levels following vaccination varies, ranging from 24% to 97% [25], and depends on epigenetic factors [15]. Differences in the percentage of seronegative children 1–10 years old according to increasing age were confirmed by a study conducted in Ghana, where children were vaccinated at 6, 10 and 14 weeks of life with DTPw-HepB-Hib, Panacea Biotech ltd, India. 12.1% were seronegative one to six months after the receipt of the last dose, 21.7% three years after, and 58.3% five years post vaccination [26]. Similarly, among western Brazilian Amazon children aged 2 to 5 years (four doses, the first a monovalent given at birth and the remaining three pentavalent vaccines given at 2, 4 and 6 months, 10μg), 32% subjects had anti-HBs titer < 10 IU/L [27]. By contrast, 44% of Senegalese children vaccinated in infancy with a monovalent recombinant vaccine were seronegative, including 20.9% under the age of five, 66.3% of 5- to 10-year-olds and 59% of children above the age of 10 years [28]. On the other hand, a higher anti-HBs titer was found in Chinese adolescents aged 17–20 years who were vaccinated with the Chinese hamster ovary (CHO)-derived HB vaccine (HebB), 10 μg/mL at the age of 0, 1, 6 months, of whom only 25.5% had anti-HBs < 10 mIU/mL [29]. Among Italian students who were vaccinated with three doses of a recombinant B vaccine at 3, 5 and 11 months of postnatal life and students who received three doses (20 μg) of the same vaccine at 12 years of age, the group of individuals vaccinated in infancy had significantly higher prevalence of low anti-HBs titer < 10 IU/L in comparison to students vaccinated at 12 years (23% vs 12.1%) [30]. Another study demonstrated that 60% of Micronesian adolescents who were vaccinated against HB at birth (Recombivax 5 μg at birth, and 2,5 μg at 2 and 6 months) were seronegative at the age of 15 years [31]. In the studies mentioned above, no significant differences between anti-HBs titer and sex were found.

In our study, we demonstrated a negative correlation between anti-HBs titer and age, and a weak positive correlation between anti-HBs titer and 25(OH)D concentration. Subjects with vitamin D deficiency had significantly lower anti-HBs titer. When we compared the subgroups of children according to age, we established that 25(OH)D concentration was highest in children aged 0.7–2 years. Significantly higher anti-HBs titer was found in the subgroup of the youngest children, being higher in children 3–5 years old in relation to older children. This is in line with the general knowledge that anti-HBs titers decline steadily after vaccination if no booster is administered. Considering the above it could be presumed that there is a relationship between 25(OH)D concentration and anti-HBs titer.

Analysis of the cluster pairs allowed us to assess the combined effect of age and 25(OH)D concentration on anti-HBs titer. Our analysis demonstrated that children from clusters A and B did not differ significantly in age (p = 0.891) and anti-HBs titer (p = 0.660). 25(OH)D levels in both groups differed significantly (p = 0.000) and the maximum value of vitamin D in cluster A was lower than the minimum value in cluster B. A much higher level of vitamin D in cluster B caused a stronger positive asymmetry than in A, and favoured the presence of very high anti-HBs titers. To look differently at the influence of high vitamin D values on anti-HBs titer, we created two subgroups of children from clusters A and B, in whom anti-HBs titer exceeded Q3 (374.5 mIU/mL) from cluster B. We found a considerably higher vitamin D level in cluster B in comparison to cluster A, with comparable age, and the frequency of achieving very high anti-HBs values increased only on the border of significance (p = 0.068).

Cluster E comprising the oldest children with low vitamin D level is noteworthy. A combination of both factors resulted in the lowest level of anti-HBs titer. Clusters C and E differed significantly in the level of anti-HBs titer. Its higher level in cluster C was "saved" by a young age and high vitamin D level. The pairs C *vs* D, D *vs* E and D *vs* F did not differ significantly in the level of anti-HBs titer, but they differed both in age and 25(OH)D concentration. In the analysis of six clusters of children we found that age, so time since the receipt of the last vaccine dose, was the main factor influencing a decline in anti-HBs titer in children under 12 years of age. Similar results have been obtained by other authors who revealed that anti-HBs titer declined with increasing age [26,27,30] and longer time after the receipt of the last vaccine dose [27].

A number of studies have explored the role of vitamin D in the immune response to vaccines as well as vitamin D signalling pathway polymorphisms, but the results were inconclusive [16]. It should be added that polymorphisms of vitamin A and D receptors are associated with lower antibody responses to HB vaccination [32]. Animal studies indicate the impact of vitamin D on vaccine response as an adjuvant [33–35], directly by inducing dendritic cell migration from a vaccine administration site to local lymph nodes, and by stimulating antigen-specific Th2 and B lymphocytes, thereby increasing vaccine-induced antibody production [36,37].

We found that vitamin D level was not a significant determinant of higher anti-HBs titer in children receiving hepatitis B vaccine in infancy. Available literature data on the impact of vitamin D on HB vaccine response are inconsistent as some researchers have demonstrated that higher vitamin D concentration is associated with better response to booster HB immunisation [18,38,39] and that vitamin D concentration is an independent significant negative predictor of seroconversion [39], while others have indicated a lack of association [40–42]. Some of them observed that low vitamin D concentration was associated with poorer HB vaccine response. However, they found that vitamin D supplementation following vaccination did not influence vaccine response [18]. Other authors revealed the role of genetic variants of vitamin D receptor rs1544410AA on the response to HB vaccination [32]. Published studies concern mainly adults over the age of 20, healthy [18,38,40], infected with HIV [42] or those with chronic renal disease including renal failure [32,39,41]. By contrast, our study was conducted on a homogeneous group of healthy children matched by age and sex.

Our study demonstrated that one third of children under 12 years of age had nonprotective anti-HBs titer < 10 mIU/mL and around 40% had vitamin D deficiency. We conclude that vitamin D status has no impact on anti-HBs titer in children vaccinated against hepatitis B virus in infancy. Age, so time since the receipt of the last dose of hepatitis B vaccine, is the main factor influencing a decline in anti-HBs titer.

## Supporting information

S1 Data (PDF)
