# Supplementary material for: Lack of relationship between 25-hydoxyvitamin D concentration and a titer of antibodies to hepatitis B surface antigen in children under 12 years of age
Source: PLoS One. 2022 Nov 10;17(11):e0277473. doi: 10.1371/journal.pone.0277473 (PMC9648759; doi:10.1371/journal.pone.0277473)
Supplement: S1 Data — (PDF) [file pone.0277473.s001.pdf]

| NR | Gender | Anti-HBs titer range (mIU/mL) | Anti-HBs titer, mIU/mL | 25(OH)D, ng/mL | 25(OH)D range (ng/mL) | Age, years | Age subgroups (years) | Clusters |
|---|---|---|---|---|---|---|---|---|
| 1 | Men | 10-100 IU/l | 71,2 | 12,70 | <20 | >1 | 0.7-2 | A |
| 2 | Women | 10-100 IU/l | 61,5 | 17,80 | <20 | >2 | 3-5 | A |
| 3 | Women | 10-100 IU/l | 84,7 | 18,50 | <20 | >1 | 0.7-2 | A |
| 4 | Men | 10-100 IU/l | 61,3 | 18,50 | <20 | >1 | 0.7-2 | A |
| 5 | Men | 10-100 IU/l | 85,0 | 18,50 | <20 | >2 | 3-5 | A |
| 6 | Women | 10-100 IU/l | 25,5 | 18,70 | <20 | >2 | 3-5 | A |
| 7 | Men | 10-100 IU/l | 55,5 | 19,20 | <20 | >1 | 0.7-2 | A |
| 8 | Women | 10-100 IU/l | 33,6 | 19,20 | <20 | >3 | 3-5 | A |
| 9 | Women | 10-100 IU/l | 79,8 | 19,30 | <20 | >1 | 0.7-2 | A |
| 10 | Women | 10-100 IU/l | 82,6 | 19,30 | <20 | >1 | 0.7-2 | A |
| 11 | Women | 10-100 IU/l | 24,1 | 19,30 | <20 | >2 | 3-5 | A |
| 12 | Men | 10-100 IU/l | 71,4 | 19,50 | <20 | >1 | 0.7-2 | A |
| 13 | Women | 10-100 IU/l | 72,5 | 19,60 | <20 | >1 | 0.7-2 | A |
| 14 | Women | 10-100 IU/l | 22,7 | 19,70 | <20 | >1 | 0.7-2 | A |
| 15 | Men | 10-100 IU/l | 16,0 | 19,90 | <20 | >3 | 3-5 | A |
| 16 | Men | 10-100 IU/l | 23,7 | 20,00 | [20-30) | >2 | 3-5 | A |
| 17 | Men | < 10 IU/l | 6,9 | 10,10 | <20 | >2 | 3-5 | A |
| 18 | Men | < 10 IU/l | 0,4 | 12,40 | <20 | >2 | 3-5 | A |
| 19 | Men | < 10 IU/l | 5,6 | 14,60 | <20 | >2 | 3-5 | A |
| 20 | Men | < 10 IU/l | 0,9 | 16,90 | <20 | >2 | 3-5 | A |
| 21 | Men | < 10 IU/l | 1,1 | 19,70 | <20 | >2 | 3-5 | A |
| 22 | Men | 100-1000 IU/l | 108,4 | 12,30 | <20 | >1 | 0.7-2 | A |
| 23 | Women | 100-1000 IU/l | 264,4 | 14,00 | <20 | >3 | 3-5 | A |
| 24 | Men | 100-1000 IU/l | 124,4 | 17,50 | <20 | >2 | 3-5 | A |
| 25 | Men | 100-1000 IU/l | 224,7 | 18,00 | <20 | >1 | 0.7-2 | A |
| 26 | Women | 100-1000 IU/l | 114,5 | 18,30 | <20 | >2 | 3-5 | A |
| 27 | Men | 100-1000 IU/l | 299,2 | 19,30 | <20 | >1 | 0.7-2 | A |
| 28 | Women | 100-1000 IU/l | 120,1 | 20,00 | [20-30) | >2 | 3-5 | A |
| 29 | Men | 10-100 IU/l | 20,0 | 21,30 | [20-30) | >2 | 3-5 | A |
| 30 | Men | 10-100 IU/l | 22,7 | 21,90 | [20-30) | >2 | 3-5 | A |
| 31 | Men | 10-100 IU/l | 86,3 | 23,00 | [20-30) | >1 | 0.7-2 | A |
| 32 | Men | 10-100 IU/l | 19,6 | 23,00 | [20-30) | >2 | 3-5 | A |
| 33 | Men | 10-100 IU/l | 21,5 | 23,50 | [20-30) | >2 | 3-5 | A |
| 34 | Women | 10-100 IU/l | 75,5 | 23,80 | [20-30) | >2 | 3-5 | A |
| 35 | Men | 10-100 IU/l | 34,9 | 23,90 | [20-30) | >1 | 0.7-2 | A |
| 36 | Men | 10-100 IU/l | 73,9 | 25,30 | [20-30) | >2 | 3-5 | A |
| 37 | Women | 10-100 IU/l | 87,3 | 25,50 | [20-30) | >1 | 0.7-2 | A |
| 38 | Women | 10-100 IU/l | 12,2 | 25,70 | [20-30) | .2 | 3-5 | A |
| 39 | Men | 10-100 IU/l | 57,9 | 26,10 | [20-30) | >1 | 0.7-2 | A |
| 40 | Women | 10-100 IU/l | 11,2 | 26,70 | [20-30) | >2 | 3-5 | A |
| 41 | Men | 10-100 IU/l | 30,8 | 26,90 | [20-30) | >2 | 3-5 | A |
| 42 | Women | 10-100 IU/l | 21,0 | 27,10 | [20-30) | >2 | 3-5 | A |
| 43 | Women | 100-1000 IU/l | 280,7 | 21,80 | [20-30) | >2 | 3-5 | A |
| 44 | Men | 100-1000 IU/l | 123,1 | 21,90 | [20-30) | >1 | 0.7-2 | A |
| 45 | Women | 100-1000 IU/l | 110,9 | 22,10 | [20-30) | >2 | 3-5 | A |
| 46 | Women | 100-1000 IU/l | 360,4 | 22,80 | [20-30) | .0.7 | 0.7-2 | A |
| 47 | Men | 100-1000 IU/l | 835,7 | 22,90 | [20-30) | >1 | 0.7-2 | A |
| 48 | Men | 100-1000 IU/l | 508,2 | 23,00 | [20-30) | >2 | 3-5 | A |
| 49 | Men | 100-1000 IU/l | 588,4 | 23,10 | [20-30) | >2 | 3-5 | A |
| 50 | Men | 100-1000 IU/l | 114,7 | 23,20 | [20-30) | >1 | 0.7-2 | A |
| 51 | Women | 100-1000 IU/l | 391,7 | 24,40 | [20-30) | >1 | 0.7-2 | A |
| 52 | Women | 100-1000 IU/l | 238,4 | 24,60 | [20-30) | >0.7 | 0.7-2 | A |
| 53 | Men | 100-1000 IU/l | 132,4 | 24,80 | [20-30) | >2 | 3-5 | A |
| 54 | Men | 100-1000 IU/l | 191,2 | 24,90 | [20-30) | >1 | 0.7-2 | A |
| 55 | Women | 100-1000 IU/l | 461,9 | 25,40 | [20-30) | >2 | 3-5 | A |
| 56 | Men | 100-1000 IU/l | 304,9 | 25,50 | [20-30) | >1 | 0.7-2 | A |
| 57 | Men | 100-1000 IU/l | 734,9 | 25,90 | [20-30) | >0.7 | 0.7-2 | A |
| 58 | Men | 100-1000 IU/l | 912,0 | 26,40 | [20-30) | >1 | 0.7-2 | A |
| 59 | Men | 100-1000 IU/l | 331,0 | 27,30 | [20-30) | >1 | 0.7-2 | A |
| 60 | Men | 10-100 IU/l | 73,6 | 27,80 | [20-30) | >1 | 0.7-2 | B |
| 61 | Men | 10-100 IU/l | 21,2 | 27,80 | [20-30) | >2 | 3-5 | B |
| 62 | Men | 10-100 IU/l | 62,1 | 28,50 | [20-30) | >1 | 0.7-2 | B |
| 63 | Women | 10-100 IU/l | 38,0 | 29,30 | [20-30) | >1 | 0.7-2 | B |
| 64 | Women | < 10 IU/l | 8,6 | 29,20 | [20-30) | >2 | 3-5 | B |
| 65 | Men | < 10 IU/l | 9,0 | 29,50 | [20-30) | >2 | 3-5 | B |
| 66 | Women | 100-1000 IU/l | 651,9 | 28,10 | [20-30) | >2 | 3-5 | B |
| 67 | Women | 100-1000 IU/l | 230,5 | 28,30 | [20-30) | >1 | 0.7-2 | B |
| 68 | Women | 100-1000 IU/l | 588,8 | 28,30 | [20-30) | >2 | 3-5 | B |
| 69 | Men | 100-1000 IU/l | 752,1 | 28,70 | [20-30) | >0.7 | 0.7-2 | B |
| 70 | Women | 100-1000 IU/l | 354,4 | 28,90 | [20-30) | >1 | 0.7-2 | B |
| 71 | Women | 100-1000 IU/l | 761,2 | 29,10 | [20-30) | >1 | 0.7-2 | B |
| 72 | Women | 100-1000 IU/l | 238,6 | 29,50 | [20-30) | >2 | 3-5 | B |
| 73 | Men | 100-1000 IU/l | 181,1 | 29,80 | [20-30) | >2 | 3-5 | B |
| 74 | Women | 100-1000 IU/l | 164,5 | 29,90 | [20-30) | >2 | 3-5 | B |
| 75 | Women | 10-100 IU/l | 41,3 | 51,40 | >=30 | >1 | 0.7-2 | B |
| 76 | Women | 10-100 IU/l | 87,5 | 30,00 | >=30 | >1 | 0.7-2 | B |
| 77 | Men | 10-100 IU/l | 45,9 | 30,80 | >=30 | >2 | 3-5 | B |
| 78 | Women | 10-100 IU/l | 25,1 | 31,50 | >=30 | >2 | 3-5 | B |
| 79 | Men | 10-100 IU/l | 75,7 | 32,00 | >=30 | >2 | 3-5 | B |
| 80 | Men | 10-100 IU/l | 41,8 | 33,30 | >=30 | >3 | 3-5 | B |
| 81 | Men | 10-100 IU/l | 14,1 | 34,10 | >=30 | >3 | 3-5 | B |
| 82 | Men | 10-100 IU/l | 60,5 | 34,60 | >=30 | >2 | 3-5 | B |
| 83 | Women | 10-100 IU/l | 32,7 | 35,00 | >=30 | >1 | 0.7-2 | B |
| 84 | Women | 10-100 IU/l | 26,2 | 35,10 | >=30 | >1 | 0.7-2 | B |
| 85 | Women | 10-100 IU/l | 55,8 | 36,30 | >=30 | >1 | 0.7-2 | B |
| 86 | Men | 10-100 IU/l | 20,8 | 38,50 | >=30 | >2 | 3-5 | B |
| 87 | Women | 10-100 IU/l | 44,4 | 39,00 | >=30 | >0.7 | 0.7-2 | B |
| 88 | Men | 10-100 IU/l | 29,7 | 40,40 | >=30 | >0.7 | 0.7-2 | B |

| | | | | | | | | |
|---|---|---|---|---|---|---|---|---|
| 89 | Men | 10-100 IU/l | 29,8 | 41,30 | >=30 | >3 | 3-5 | B |
| 90 | Men | 10-100 IU/l | 88,5 | 43,00 | >=30 | >1 | 0.7-2 | B |
| 91 | Women | 10-100 IU/l | 48,8 | 48,60 | >=30 | >2 | 3-5 | B |
| 92 | Men | < 10 IU/l | 4,5 | 30,50 | >=30 | >2 | 3-5 | B |
| 93 | Women | < 10 IU/l | 1,1 | 32,00 | >=30 | >2 | 3-5 | B |
| 94 | Men | < 10 IU/l | 2,8 | 35,10 | >=30 | >4 | 3-5 | B |
| 95 | Men | < 10 IU/l | 6,1 | 35,20 | >=30 | >2 | 3-5 | B |
| 96 | Women | < 10 IU/l | 8,3 | 35,70 | >=30 | >3 | 3-5 | B |
| 97 | Men | < 10 IU/l | 0,5 | 37,80 | >=30 | >2 | 3-5 | B |
| 98 | Women | < 10 IU/l | 2,1 | 41,90 | >=30 | >4 | 3-5 | B |
| 99 | Men | < 10 IU/l | 6,6 | 49,90 | >=30 | >0.7 | 0.7-2 | B |
| 100 | Men | 100-1000 IU/l | 852,6 | 30,00 | >=30 | >2 | 3-5 | B |
| 101 | Men | 100-1000 IU/l | 143,0 | 30,40 | >=30 | >2 | 3-5 | B |
| 102 | Men | 100-1000 IU/l | 153,1 | 31,00 | >=30 | >1 | 0.7-2 | B |
| 103 | Men | 100-1000 IU/l | 869,6 | 31,40 | >=30 | >1 | 0.7-2 | B |
| 104 | Women | 100-1000 IU/l | 185,9 | 31,80 | >=30 | >3 | 3-5 | B |
| 105 | Men | 100-1000 IU/l | 880,0 | 32,40 | >=30 | >2 | 3-5 | B |
| 106 | Men | 100-1000 IU/l | 181,5 | 33,00 | >=30 | >1 | 0.7-2 | B |
| 107 | Men | 100-1000 IU/l | 760,4 | 33,70 | >=30 | >2 | 3-5 | B |
| 108 | Men | 100-1000 IU/l | 190,5 | 34,00 | >=30 | >0.7 | 0.7-2 | B |
| 109 | Men | 100-1000 IU/l | 738,5 | 34,30 | >=30 | >3 | 3-5 | B |
| 110 | Men | 100-1000 IU/l | 492,1 | 34,60 | >=30 | >1 | 0.7-2 | B |
| 111 | Men | 100-1000 IU/l | 399,2 | 35,20 | >=30 | >2 | 3-5 | B |
| 112 | Women | 100-1000 IU/l | 733,7 | 35,30 | >=30 | >0.7 | 0.7-2 | B |
| 113 | Women | 100-1000 IU/l | 163,4 | 38,90 | >=30 | >2 | 3-5 | B |
| 114 | Women | 100-1000 IU/l | 394,5 | 39,80 | >=30 | >1 | 0.7-2 | B |
| 115 | Men | 100-1000 IU/l | 410,1 | 41,60 | >=30 | >0.7 | 0.7-2 | B |
| 116 | Women | 10-100 IU/l | 10,2 | 18,30 | <20 | >3 | 3-5 | C |
| 117 | Women | 10-100 IU/l | 25,7 | 19,00 | <20 | >3 | 3-5 | C |
| 118 | Men | < 10 IU/l | 5,4 | 19,00 | <20 | >4 | 3-5 | C |
| 119 | Men | < 10 IU/l | 0,4 | 19,40 | <20 | >4 | 3-5 | C |
| 120 | Women | < 10 IU/l | 0,0 | 19,80 | <20 | >4 | 3-5 | C |
| 121 | Men | < 10 IU/l | 4,6 | 20,00 | [20-30) | >3 | 3-5 | C |
| 122 | Women | < 10 IU/l | 7,2 | 20,00 | [20-30) | >4 | 3-5 | C |
| 123 | Men | 100-1000 IU/l | 199,6 | 18,50 | <20 | >3 | 3-5 | C |
| 124 | Men | 100-1000 IU/l | 219,2 | 19,30 | <20 | >4 | 3-5 | C |
| 125 | Men | 100-1000 IU/l | 140,2 | 19,50 | <20 | >3 | 3-5 | C |
| 126 | Men | 10-100 IU/l | 26,5 | 20,30 | [20-30) | >3 | 3-5 | C |
| 127 | Men | 10-100 IU/l | 26,3 | 20,80 | [20-30) | >3 | 3-5 | C |
| 128 | Men | 10-100 IU/l | 16,4 | 20,90 | [20-30) | >4 | 3-5 | C |
| 129 | Men | 10-100 IU/l | 24,2 | 21,00 | [20-30) | >4 | 3-5 | C |
| 130 | Women | 10-100 IU/l | 34,7 | 21,40 | [20-30) | >5 | 6-11 | C |
| 131 | Men | 10-100 IU/l | 17,2 | 22,60 | [20-30) | >4 | 3-5 | C |
| 132 | Women | 10-100 IU/l | 27,2 | 22,80 | [20-30) | >5 | 6-11 | C |
| 133 | Women | 10-100 IU/l | 13,3 | 22,90 | [20-30) | >3 | 3-5 | C |
| 134 | Men | 10-100 IU/l | 43,9 | 22,90 | [20-30) | >4 | 3-5 | C |
| 135 | Men | 10-100 IU/l | 44,7 | 23,20 | [20-30) | >3 | 3-5 | C |
| 136 | Women | 10-100 IU/l | 75,3 | 24,50 | [20-30) | >3 | 3-5 | C |
| 137 | Men | 10-100 IU/l | 37,9 | 24,60 | [20-30) | >3 | 3-5 | C |
| 138 | Men | 10-100 IU/l | 19,6 | 24,60 | [20-30) | >3 | 3-5 | C |
| 139 | Men | 10-100 IU/l | 50,5 | 24,70 | [20-30) | >3 | 3-5 | C |
| 140 | Women | 10-100 IU/l | 42,9 | 24,90 | [20-30) | >4 | 3-5 | C |
| 141 | Men | 10-100 IU/l | 49,2 | 25,80 | [20-30) | >4 | 3-5 | C |
| 142 | Women | 10-100 IU/l | 32,5 | 25,90 | [20-30) | >3 | 3-5 | C |
| 143 | Women | 10-100 IU/l | 12,4 | 26,10 | [20-30) | >3 | 3-5 | C |
| 144 | Women | 10-100 IU/l | 53,8 | 26,40 | [20-30) | >3 | 3-5 | C |
| 145 | Men | 10-100 IU/l | 11,2 | 26,50 | [20-30) | >4 | 3-5 | C |
| 146 | Men | 10-100 IU/l | 33,5 | 26,60 | [20-30) | >3 | 3-5 | C |
| 147 | Men | 10-100 IU/l | 98,6 | 26,70 | [20-30) | >3 | 3-5 | C |
| 148 | Men | 10-100 IU/l | 47,2 | 27,00 | [20-30) | >2 | 3-5 | C |
| 149 | Women | 10-100 IU/l | 53,7 | 27,10 | [20-30) | >4 | 3-5 | C |
| 150 | Women | 10-100 IU/l | 14,2 | 27,20 | [20-30) | >3 | 3-5 | C |
| 151 | Men | 10-100 IU/l | 49,5 | 27,90 | [20-30) | >3 | 3-5 | C |
| 152 | Women | 10-100 IU/l | 60,2 | 28,80 | [20-30) | >4 | 3-5 | C |
| 153 | Men | 10-100 IU/l | 66,4 | 29,90 | [20-30) | >3 | 3-5 | C |
| 154 | Women | < 10 IU/l | 8,1 | 20,60 | [20-30) | >3 | 3-5 | C |
| 155 | Men | < 10 IU/l | 0,7 | 21,00 | [20-30) | >3 | 3-5 | C |
| 156 | Men | < 10 IU/l | 3,7 | 21,40 | [20-30) | >5 | 6-11 | C |
| 157 | Women | < 10 IU/l | 0,7 | 22,30 | [20-30) | >3 | 3-5 | C |
| 158 | Women | < 10 IU/l | 4,0 | 22,50 | [20-30) | >4 | 3-5 | C |
| 159 | Women | < 10 IU/l | 9,1 | 22,50 | [20-30) | >4 | 3-5 | C |
| 160 | Women | < 10 IU/l | 2,4 | 22,90 | [20-30) | >4 | 3-5 | C |
| 161 | Men | < 10 IU/l | 3,7 | 22,90 | [20-30) | >4 | 3-5 | C |
| 162 | Men | < 10 IU/l | 6,9 | 23,90 | [20-30) | >3 | 3-5 | C |
| 163 | Men | < 10 IU/l | 3,9 | 24,00 | [20-30) | >4 | 3-5 | C |
| 164 | Men | < 10 IU/l | 6,7 | 24,60 | [20-30) | >3 | 3-5 | C |
| 165 | Men | < 10 IU/l | 6,9 | 25,00 | [20-30) | >4 | 3-5 | C |
| 166 | Women | < 10 IU/l | 3,2 | 26,90 | [20-30) | >2 | 3-5 | C |
| 167 | Women | < 10 IU/l | 2,4 | 26,90 | [20-30) | >4 | 3-5 | C |
| 168 | Men | < 10 IU/l | 2,0 | 27,50 | [20-30) | >2 | 3-5 | C |
| 169 | Men | < 10 IU/l | 6,5 | 29,00 | [20-30) | >3 | 3-5 | C |
| 170 | Men | 100-1000 IU/l | 416,8 | 20,10 | [20-30) | >3 | 3-5 | C |
| 171 | Women | 100-1000 IU/l | 743,9 | 20,40 | [20-30) | >4 | 3-5 | C |
| 172 | Men | 100-1000 IU/l | 163,0 | 20,80 | [20-30) | >5 | 6-11 | C |
| 173 | Women | 100-1000 IU/l | 114,1 | 22,00 | [20-30) | >3 | 3-5 | C |
| 174 | Men | 100-1000 IU/l | 167,6 | 23,00 | [20-30) | >3 | 3-5 | C |
| 175 | Men | 100-1000 IU/l | 106,6 | 24,00 | [20-30) | >4 | 3-5 | C |
| 176 | Women | 100-1000 IU/l | 444,8 | 25,40 | [20-30) | >3 | 3-5 | C |
| 177 | Women | 100-1000 IU/l | 182,5 | 25,40 | [20-30) | >4 | 3-5 | C |
| 178 | Men | 100-1000 IU/l | 309,9 | 25,60 | [20-30) | >3 | 3-5 | C |

| | | | | | | | | |
|---|---|---|---|---|---|---|---|---|
| 179 | Women | 100-1000 IU/l | 163,9 | 27,50 | [20-30) | >3 | 3-5 | C |
| 180 | Men | 100-1000 IU/l | 212,6 | 28,70 | [20-30) | >3 | 3-5 | C |
| 181 | Women | 100-1000 IU/l | 171,8 | 29,20 | [20-30) | >3 | 3-5 | C |
| 182 | Men | 100-1000 IU/l | 588,6 | 29,80 | [20-30) | >5 | 6-11 | C |
| 183 | Women | 10-100 IU/l | 12,4 | 30,60 | >=30 | >3 | 3-5 | C |
| 184 | Women | 10-100 IU/l | 21,2 | 30,60 | >=30 | >4 | 3-5 | C |
| 185 | Men | 100-1000 IU/l | 290,0 | 32,80 | >=30 | >4 | 3-5 | C |
| 186 | Men | 10-100 IU/l | 47,1 | 6,70 | <20 | >5 | 6-11 | D |
| 187 | Women | 10-100 IU/l | 39,3 | 8,10 | <20 | >3 | 3-5 | D |
| 188 | Men | 10-100 IU/l | 32,9 | 8,10 | <20 | >5 | 6-11 | D |
| 189 | Women | 10-100 IU/l | 14,6 | 9,20 | <20 | >3 | 3-5 | D |
| 190 | Women | 10-100 IU/l | 36,9 | 9,20 | <20 | >3 | 3-5 | D |
| 191 | Women | 10-100 IU/l | 83,6 | 9,60 | <20 | >5 | 6-11 | D |
| 192 | Women | 10-100 IU/l | 47,4 | 9,60 | <20 | >6 | 6-11 | D |
| 193 | Men | < 10 IU/l | 2,3 | 8,40 | <20 | >3 | 3-5 | D |
| 194 | Women | < 10 IU/l | 0,0 | 8,60 | <20 | >4 | 3-5 | D |
| 195 | Men | < 10 IU/l | 0,0 | 9,10 | <20 | >6 | 6-11 | D |
| 196 | Men | < 10 IU/l | 0,0 | 9,60 | <20 | >4 | 3-5 | D |
| 197 | Men | 100-1000 IU/l | 133,0 | 8,80 | <20 | >6 | 6-11 | D |
| 198 | Women | 10-100 IU/l | 11,6 | 10,30 | <20 | >4 | 3-5 | D |
| 199 | Men | 10-100 IU/l | 17,5 | 10,80 | <20 | >5 | 6-11 | D |
| 200 | Women | 10-100 IU/l | 11,7 | 11,50 | <20 | >4 | 3-5 | D |
| 201 | Women | 10-100 IU/l | 24,6 | 11,60 | <20 | >6 | 6-11 | D |
| 202 | Men | 10-100 IU/l | 13,8 | 12,20 | <20 | >3 | 3-5 | D |
| 203 | Men | 10-100 IU/l | 36,6 | 12,30 | <20 | >5 | 6-11 | D |
| 204 | Men | 10-100 IU/l | 91,8 | 12,90 | <20 | >4 | 3-5 | D |
| 205 | Women | 10-100 IU/l | 22,5 | 13,20 | <20 | >3 | 3-5 | D |
| 206 | Women | 10-100 IU/l | 21,6 | 13,30 | <20 | >4 | 3-5 | D |
| 207 | Men | 10-100 IU/l | 84,0 | 13,50 | <20 | >3 | 3-5 | D |
| 208 | Men | 10-100 IU/l | 17,8 | 13,50 | <20 | >4 | 3-5 | D |
| 209 | Men | 10-100 IU/l | 87,9 | 13,50 | <20 | >5 | 6-11 | D |
| 210 | Men | 10-100 IU/l | 28,5 | 13,90 | <20 | >6 | 6-11 | D |
| 211 | Women | 10-100 IU/l | 10,9 | 14,00 | <20 | >7 | 6-11 | D |
| 212 | Men | 10-100 IU/l | 13,2 | 14,20 | <20 | >6 | 6-11 | D |
| 213 | Men | 10-100 IU/l | 39,8 | 14,30 | <20 | >6 | 6-11 | D |
| 214 | Men | 10-100 IU/l | 61,9 | 15,30 | <20 | >4 | 3-5 | D |
| 215 | Men | 10-100 IU/l | 58,1 | 15,80 | <20 | >3 | 3-5 | D |
| 216 | Women | 10-100 IU/l | 12,0 | 15,80 | <20 | >5 | 6-11 | D |
| 217 | Men | 10-100 IU/l | 54,3 | 15,90 | <20 | >4 | 3-5 | D |
| 218 | Women | 10-100 IU/l | 34,6 | 16,10 | <20 | >6 | 6-11 | D |
| 219 | Men | 10-100 IU/l | 28,2 | 16,20 | <20 | >3 | 3-5 | D |
| 220 | Women | 10-100 IU/l | 67,5 | 16,30 | <20 | >3 | 3-5 | D |
| 221 | Women | 10-100 IU/l | 11,0 | 16,30 | <20 | >4 | 3-5 | D |
| 222 | Women | 10-100 IU/l | 13,7 | 16,40 | <20 | >3 | 3-5 | D |
| 223 | Men | 10-100 IU/l | 43,1 | 16,50 | <20 | >6 | 6-11 | D |
| 224 | Women | 10-100 IU/l | 94,0 | 16,60 | <20 | >6 | 6-11 | D |
| 225 | Women | 10-100 IU/l | 15,1 | 17,30 | <20 | >4 | 3-5 | D |
| 226 | Men | 10-100 IU/l | 80,1 | 17,50 | <20 | >4 | 3-5 | D |
| 227 | Men | 10-100 IU/l | 54,0 | 18,00 | <20 | >5 | 6-11 | D |
| 228 | Men | 10-100 IU/l | 67,2 | 18,00 | <20 | >6 | 6-11 | D |
| 229 | Men | 10-100 IU/l | 14,4 | 18,30 | <20 | >5 | 6-11 | D |
| 230 | Women | 10-100 IU/l | 27,4 | 18,40 | <20 | >5 | 6-11 | D |
| 231 | Women | < 10 IU/l | 2,0 | 11,40 | <20 | >4 | 3-5 | D |
| 232 | Men | < 10 IU/l | 0,7 | 11,60 | <20 | >6 | 6-11 | D |
| 233 | Men | < 10 IU/l | 2,9 | 11,60 | <20 | >6 | 6-11 | D |
| 234 | Men | < 10 IU/l | 4,1 | 11,60 | <20 | >7 | 6-11 | D |
| 235 | Men | < 10 IU/l | 2,0 | 11,80 | <20 | >5 | 6-11 | D |
| 236 | Women | < 10 IU/l | 1,1 | 12,70 | <20 | >4 | 3-5 | D |
| 237 | Women | < 10 IU/l | 0,4 | 12,70 | <20 | >4 | 3-5 | D |
| 238 | Men | < 10 IU/l | 9,4 | 12,80 | <20 | >4 | 3-5 | D |
| 239 | Women | < 10 IU/l | 4,9 | 13,00 | <20 | >6 | 6-11 | D |
| 240 | Women | < 10 IU/l | 0,4 | 14,10 | <20 | >6 | 6-11 | D |
| 241 | Men | < 10 IU/l | 2,6 | 14,80 | <20 | >5 | 6-11 | D |
| 242 | Women | < 10 IU/l | 1,0 | 15,50 | <20 | >4 | 3-5 | D |
| 243 | Men | < 10 IU/l | 2,0 | 15,80 | <20 | >5 | 6-11 | D |
| 244 | Men | < 10 IU/l | 0,4 | 16,00 | <20 | >6 | 6-11 | D |
| 245 | Women | < 10 IU/l | 1,6 | 16,80 | <20 | >6 | 6-11 | D |
| 246 | Men | < 10 IU/l | 0,9 | 16,90 | <20 | >5 | 6-11 | D |
| 247 | Women | < 10 IU/l | 3,6 | 17,20 | <20 | >3 | 3-5 | D |
| 248 | Women | < 10 IU/l | 9,2 | 18,50 | <20 | >4 | 3-5 | D |
| 249 | Men | 100-1000 IU/l | 127,9 | 11,40 | <20 | >4 | 3-5 | D |
| 250 | Men | 100-1000 IU/l | 206,1 | 11,80 | <20 | >4 | 3-5 | D |
| 251 | Women | 10-100 IU/l | 100,0 | 11,90 | <20 | >3 | 3-5 | D |
| 252 | Women | 100-1000 IU/l | 213,4 | 12,20 | <20 | >3 | 3-5 | D |
| 253 | Women | 100-1000 IU/l | 122,6 | 12,90 | <20 | >5 | 6-11 | D |
| 254 | Men | 100-1000 IU/l | 121,5 | 14,50 | <20 | >5 | 6-11 | D |
| 255 | Men | 100-1000 IU/l | 291,7 | 15,70 | <20 | >6 | 6-11 | D |
| 256 | Men | 100-1000 IU/l | 141,1 | 17,20 | <20 | >5 | 6-11 | D |
| 257 | Men | 100-1000 IU/l | 123,0 | 17,40 | <20 | >4 | 3-5 | D |
| 258 | Men | 100-1000 IU/l | 321,5 | 18,20 | <20 | >4 | 3-5 | D |
| 259 | Men | 10-100 IU/l | 10,5 | 8,30 | <20 | >8 | 6-11 | E |
| 260 | Men | 10-100 IU/l | 12,7 | 8,30 | <20 | >10 | 6-11 | E |
| 261 | Men | 10-100 IU/l | 22,4 | 8,80 | <20 | >9 | 6-11 | E |
| 262 | Men | 10-100 IU/l | 11,9 | 11,90 | <20 | >8 | 6-11 | E |
| 263 | Men | 10-100 IU/l | 26,7 | 12,70 | <20 | >7 | 6-11 | E |
| 264 | Women | 10-100 IU/l | 42,7 | 13,50 | <20 | >8 | 6-11 | E |
| 265 | Men | 10-100 IU/l | 10,8 | 14,10 | <20 | >8 | 6-11 | E |
| 266 | Men | 10-100 IU/l | 12,2 | 15,30 | <20 | >10 | 6-11 | E |
| 267 | Men | 10-100 IU/l | 19,4 | 15,50 | <20 | >11 | 6-11 | E |
| 268 | Women | 10-100 IU/l | 11,1 | 16,70 | <20 | >7 | 6-11 | E |

| | | | | | | | | |
|---|---|---|---|---|---|---|---|---|
| 269 | Women | 10-100 IU/l | 43,9 | 17,60 | <20 | >9 | 6-11 | E |
| 270 | Men | 10-100 IU/l | 21,8 | 17,80 | <20 | >7 | 6-11 | E |
| 271 | Men | 10-100 IU/l | 19,4 | 19,20 | <20 | >9 | 6-11 | E |
| 272 | Women | < 10 IU/l | 3,5 | 12,10 | <20 | >10 | 6-11 | E |
| 273 | Women | < 10 IU/l | 5,3 | 12,80 | <20 | >9 | 6-11 | E |
| 274 | Men | < 10 IU/l | 0,5 | 13,80 | <20 | >10 | 6-11 | E |
| 275 | Men | < 10 IU/l | 1,6 | 14,00 | <20 | >7 | 6-11 | E |
| 276 | Men | < 10 IU/l | 5,6 | 14,90 | <20 | >8 | 6-11 | E |
| 277 | Women | < 10 IU/l | 2,0 | 14,90 | <20 | >11 | 6-11 | E |
| 278 | Men | < 10 IU/l | 5,2 | 16,80 | <20 | >9 | 6-11 | E |
| 279 | Men | < 10 IU/l | 1,6 | 17,20 | <20 | >10 | 6-11 | E |
| 280 | Women | < 10 IU/l | 9,9 | 17,50 | <20 | >9 | 6-11 | E |
| 281 | Women | < 10 IU/l | 3,3 | 17,60 | <20 | >7 | 6-11 | E |
| 282 | Women | < 10 IU/l | 1,3 | 18,10 | <20 | >11 | 6-11 | E |
| 283 | Women | < 10 IU/l | 3,5 | 18,10 | <20 | >11 | 6-11 | E |
| 284 | Women | < 10 IU/l | 0,5 | 18,60 | <20 | >7 | 6-11 | E |
| 285 | Women | < 10 IU/l | 2,0 | 18,70 | <20 | >9 | 6-11 | E |
| 286 | Women | < 10 IU/l | 5,8 | 19,40 | <20 | >8 | 6-11 | E |
| 287 | Men | 100-1000 IU/l | 256,9 | 11,30 | <20 | >7 | 6-11 | E |
| 288 | Men | 100-1000 IU/l | 149,2 | 16,70 | <20 | >9 | 6-11 | E |
| 289 | Men | 10-100 IU/l | 28,8 | 20,30 | [20-30) | >7 | 6-11 | E |
| 290 | Men | 10-100 IU/l | 29,4 | 22,40 | [20-30) | >11 | 6-11 | E |
| 291 | Women | < 10 IU/l | 2,5 | 20,90 | [20-30) | >9 | 6-11 | E |
| 292 | Men | < 10 IU/l | 1,0 | 21,60 | [20-30) | >8 | 6-11 | E |
| 293 | Women | < 10 IU/l | 0,2 | 22,00 | [20-30) | >9 | 6-11 | E |
| 294 | Men | < 10 IU/l | 2,1 | 22,20 | [20-30) | >8 | 6-11 | E |
| 295 | Men | < 10 IU/l | 2,4 | 24,40 | [20-30) | >11 | 6-11 | E |
| 296 | Men | < 10 IU/l | 4,6 | 25,30 | [20-30) | >9 | 6-11 | E |
| 297 | Men | < 10 IU/l | 4,3 | 25,70 | [20-30) | >9 | 6-11 | E |
| 298 | Women | < 10 IU/l | 6,4 | 26,00 | [20-30) | >10 | 6-11 | E |
| 299 | Men | 100-1000 IU/l | 101,0 | 22,20 | [20-30) | >11 | 6-11 | E |
| 300 | Men | 100-1000 IU/l | 167,8 | 22,70 | [20-30) | >9 | 6-11 | E |
| 301 | Men | 100-1000 IU/l | 141,6 | 23,30 | [20-30) | >10 | 6-11 | E |
| 302 | Women | 100-1000 IU/l | 785,7 | 24,00 | [20-30) | >10 | 6-11 | E |
| 303 | Men | 100-1000 IU/l | 193,6 | 25,90 | [20-30) | >9 | 6-11 | E |
| 304 | Men | < 10 IU/l | 5,7 | 10,00 | <20 | >8 | 6-11 | E |
| 305 | Men | 10-100 IU/l | 52,3 | 18,50 | <20 | >7 | 6-11 | F |
| 306 | Men | 10-100 IU/l | 11,6 | 19,50 | <20 | >6 | 6-11 | F |
| 307 | Men | 100-1000 IU/l | 183,9 | 18,60 | <20 | >6 | 6-11 | F |
| 308 | Men | 10-100 IU/l | 54,9 | 20,30 | [20-30) | >5 | 6-11 | F |
| 309 | Men | 10-100 IU/l | 16,1 | 20,30 | [20-30) | >7 | 6-11 | F |
| 310 | Men | 10-100 IU/l | 22,8 | 20,80 | [20-30) | >6 | 6-11 | F |
| 311 | Women | 10-100 IU/l | 82,0 | 21,00 | [20-30) | >6 | 6-11 | F |
| 312 | Women | 10-100 IU/l | 48,8 | 22,10 | [20-30) | >6 | 6-11 | F |
| 313 | Men | 10-100 IU/l | 18,5 | 22,20 | [20-30) | >5 | 6-11 | F |
| 314 | Men | 10-100 IU/l | 42,0 | 22,40 | [20-30) | >5 | 6-11 | F |
| 315 | Women | 10-100 IU/l | 53,5 | 23,10 | [20-30) | >5 | 6-11 | F |
| 316 | Women | 10-100 IU/l | 32,4 | 23,40 | [20-30) | >7 | 6-11 | F |
| 317 | Women | 10-100 IU/l | 38,4 | 23,50 | [20-30) | >7 | 6-11 | F |
| 318 | Men | 10-100 IU/l | 17,3 | 24,80 | [20-30) | >7 | 6-11 | F |
| 319 | Women | 10-100 IU/l | 12,9 | 25,80 | [20-30) | >6 | 6-11 | F |
| 320 | Men | 10-100 IU/l | 64,9 | 26,30 | [20-30) | >7 | 6-11 | F |
| 321 | Men | 10-100 IU/l | 27,8 | 26,70 | [20-30) | >5 | 6-11 | F |
| 322 | Men | 10-100 IU/l | 70,1 | 26,90 | [20-30) | >5 | 6-11 | F |
| 323 | Men | 10-100 IU/l | 14,1 | 27,00 | [20-30) | >6 | 6-11 | F |
| 324 | Men | 10-100 IU/l | 11,7 | 27,40 | [20-30) | >6 | 6-11 | F |
| 325 | Women | 10-100 IU/l | 69,7 | 27,50 | [20-30) | >7 | 6-11 | F |
| 326 | Men | 10-100 IU/l | 63,0 | 27,60 | [20-30) | >5 | 6-11 | F |
| 327 | Women | 10-100 IU/l | 62,1 | 27,80 | [20-30) | >5 | 6-11 | F |
| 328 | Men | 10-100 IU/l | 30,7 | 28,60 | [20-30) | >5 | 6-11 | F |
| 329 | Women | 10-100 IU/l | 46,9 | 29,50 | [20-30) | >6 | 6-11 | F |
| 330 | Men | < 10 IU/l | 9,5 | 22,40 | [20-30) | >6 | 6-11 | F |
| 331 | Men | < 10 IU/l | 2,7 | 22,60 | [20-30) | >7 | 6-11 | F |
| 332 | Women | < 10 IU/l | 5,2 | 22,70 | [20-30) | >7 | 6-11 | F |
| 333 | Women | < 10 IU/l | 1,1 | 23,10 | [20-30) | >7 | 6-11 | F |
| 334 | Men | < 10 IU/l | 2,1 | 23,30 | [20-30) | >6 | 6-11 | F |
| 335 | Women | < 10 IU/l | 5,5 | 23,40 | [20-30) | >7 | 6-11 | F |
| 336 | Men | < 10 IU/l | 0,6 | 24,00 | [20-30) | >5 | 6-11 | F |
| 337 | Women | 10-100 IU/l | 10,0 | 24,90 | [20-30) | >5 | 6-11 | F |
| 338 | Men | < 10 IU/l | 0,2 | 24,90 | [20-30) | >6 | 6-11 | F |
| 339 | Men | < 10 IU/l | 7,7 | 25,20 | [20-30) | >7 | 6-11 | F |
| 340 | Women | < 10 IU/l | 5,2 | 26,60 | [20-30) | >5 | 6-11 | F |
| 341 | Men | < 10 IU/l | 7,2 | 26,80 | [20-30) | >5 | 6-11 | F |
| 342 | Men | < 10 IU/l | 3,6 | 28,70 | [20-30) | >6 | 6-11 | F |
| 343 | Men | < 10 IU/l | 6,6 | 29,50 | [20-30) | >5 | 6-11 | F |
| 344 | Men | 100-1000 IU/l | 247,1 | 21,90 | [20-30) | >6 | 6-11 | F |
| 345 | Women | 100-1000 IU/l | 195,4 | 22,10 | [20-30) | >7 | 6-11 | F |
| 346 | Women | 10-100 IU/l | 79,4 | 32,20 | >=30 | >6 | 6-11 | F |
| 347 | Men | < 10 IU/l | 2,9 | 31,20 | >=30 | >5 | 6-11 | F |
| 348 | Women | < 10 IU/l | 1,9 | 31,80 | >=30 | >6 | 6-11 | F |
| 349 | Men | < 10 IU/l | 2,2 | 32,00 | >=30 | >6 | 6-11 | F |
| 350 | Men | < 10 IU/l | 7,8 | 49,30 | >=30 | >9 | 6-11 | F |
| 351 | Women | 100-1000 IU/l | 257,2 | 30,00 | >=30 | >5 | 6-11 | F |
| 352 | Men | 100-1000 IU/l | 133,4 | 34,70 | >=30 | >6 | 6-11 | F |
